# Predictive Value of Inflammatory Scores for Left Atrium Thrombosis in Ischemic Stroke Without Atrial Fibrillation

**DOI:** 10.3390/medicina60122046

**Published:** 2024-12-12

**Authors:** Vedat Cicek, Sahhan Kilic, Selami Dogan, Almina Erdem, Mert Babaoglu, Irem Yilmaz, Salih Karaismail, Murat Mert Atmaca, Mert Ilker Hayiroglu, Tufan Cinar, Ulas Bagci

**Affiliations:** 1 Machine & Hybrid Intelligence Lab, Department of Radiology, Northwestern University, 737 N. Michigan Avenue Suite 1600, Chicago, IL 60611, USA; 2 Department of Cardiology, Sultan II. Abdulhamid Han Training and Research Hospital, Health Sciences University, 34668 Istanbul, Turkey; sahhanklc94@gmail.com (S.K.); alminaerdem1996@hotmail.com (A.E.); babaoglumert11@gmail.com (M.B.); iremyilmaz8039@gmail.com (I.Y.); 3 Malkara State Hospital, 59300 Tekirdag, Turkey; selamidogan61@gmail.com; 4 Department of Neurology, Sultan II. Abdulhamid Han Training and Research Hospital, Health Sciences University, 34668 Istanbul, Turkey; salih.krsml@gmail.com (S.K.); drmuratmertatmaca@hotmail.com (M.M.A.); 5 Department of Cardiology, Dr. Siyami Ersek Cardiovascular and Thoracic Surgery Research and Training Hospital, 34668 Istanbul, Turkey; mertilkerh@gmail.com; 6 School of Medicine, University of Maryland, Baltimore, MD 21201, USA; drtufancinar@gmail.com

**Keywords:** thrombosis, stroke, inflammation scores, left atrial thrombosis

## Abstract

*Background and Objectives*: Studies have shown that inflammation markers can be used as prognostic tools in predicting acute ischemic stroke. In this study, we conducted a comparison of several inflammation scores in predicting left atrial thrombosis (LAT) in patients with ischemic stroke without AF. *Materials and Methods*: In this single-center, retrospective study, we included 303 consecutive patients with ischemic stroke. Each patient underwent a transesophageal echocardiography (TEE) examination within 10 days of admission to detect the presence of LAT. To identify independent predictors of LAT, we conducted a multivariate logistic regression analysis. *Results*: In total, 303 patients who had ischemic stroke were included in the analysis. LAT was detached in 34 patients at the time of the TEE examination. The patients were categorized into two groups based on their LAT status. The Prognostic Nutritional Index (PNI), HALP score, and C-reactive Protein–Albumin Ratio (CAR) were identified as statistically significant predictors of LAT. Based on the results of the multivariate regression analysis, the CAR emerged as the only independent predictor of LAT. *Conclusions*: Among several inflammation scores, the PNI, HALP, and CAR were statistically significant predictors of LAT in ischemic stroke patients without AF. CAR was identified as the optimal score for the prediction of LAT in patients with stroke and without AF.

## 1. Introduction

Stroke is one of the main causes of morbidity and mortality globally. According to the World Stroke Organization, over 12.2 million new strokes are reported annually [1]. Statistically, one in four individuals over the age of 25 can suffer a stroke in their lifetime. Ischemic stroke constitutes approximately 80% of all stroke cases [2]. Precise identification of the etiology of ischemic stroke is critical for timely therapeutic interventions aimed at treating the root cause and preventing subsequent ischemic events. Despite this, the determination of the underlying cause is often complex and relies on clinical evaluation, imaging modalities, and other diagnostic tests. The timing of the detection resources in stroke patients is crucial for the administration of time-sensitive treatments, which significantly improve clinical outcomes and reduce disability [3].

Cardio-embolic stroke is a common and preventable cause of ischemic stroke associated with higher morbidity and mortality compared with other etiologies [4]. Previous studies have established that atrial fibrillation (AF) is the primary cause of cardio-embolic stroke, attributed to the formation of left atrial thrombi (LAT) [5,6]. However, the current literature lacks clear data on the predictors of LAT in acute ischemic stroke patients who do not have AF.

In clinical practice, transthoracic echocardiography (TTE) serves as the primary imaging technique for patients diagnosed with acute ischemic stroke. Nonetheless, TTE alone often falls short of completely ruling out all potential causes of cardio-embolism. Furthermore, current guidelines do not provide a strong recommendation regarding whether patients with normal TTE findings should undergo additional transesophageal echocardiography (TEE) to identify potential sources of cardio-embolism in cases of ischemic stroke with unclear etiology [7]. Given that TEE is a semi-invasive procedure associated with certain risks in patients who have recently experienced an ischemic stroke, it is crucial to predict those patients who are most likely to benefit from this examination in clinical practice.

Inflammation scores serve as valuable tools in predicting disease outcomes and guiding clinical decision-making across various medical fields. Their ability to quantify the inflammatory state provides insights into disease pathogenesis and helps in stratifying patients based on their risk profiles. In the literature, several studies on inflammatory scores revealed that they can be applied to predict prognosis in patients with cancer, cardiovascular diseases, and autoimmune diseases. [8]. However, it is unclear whether they can be used to predict LAT in acute ischemic stroke patients without AF. Our primary aim was to determine which score provides the most accurate prediction for LAT in acute ischemic stroke patients without AF.

## 2. Methods

### 2.1. Patients

This single-center, retrospective study enrolled 303 consecutive patients presenting with acute ischemic stroke who underwent TEE for suspected cardio-embolic etiology at a tertiary hospital center between 2017 and 2023. Patients were excluded if they had a history of atrial fibrillation (AF) (either paroxysmal or persistent), an ischemic stroke with an identifiable non-cardio-embolic cause, or confirmed thrombosis in the left ventricle or right atrium. For all included patients, ischemic stroke or transient ischemic attack (TIA) was diagnosed based on findings from brain imaging studies. To exclude strokes of atherothrombotic origin, all patients also underwent either magnetic resonance angiography or computed tomography angiography, as clinically indicated. In accordance with hospital protocol, an experienced neurologist assessed each patient upon admission for clinical signs and symptoms of ischemic stroke, and 24–72 h of Holter monitoring was systematically performed to screen for potential arrhythmias, including AF. Clinical data for all patients were retrospectively collected from the hospital’s electronic medical records. The study received ethical approval from the local independent ethics committee (approval number: HNEAH/KAEK/2019/KK/171) and was conducted in line with Good Clinical Practice guidelines and the principles of the Declaration of Helsinki.

### 2.2. Inflammation Scores

This study utilized various inflammation scores defined by specific cutoff points. The Neutrophil-to-Lymphocyte Ratio (NLR) was categorized with thresholds of ≥3 [9] and ≥4 [10]. The modified Glasgow Prognostic Score (mGPS) was determined as follows: CRP ≤ 8 mg/L and albumin ≥ 35 g/L corresponded to score 0; one abnormal parameter equated to score 1; both abnormal parameters resulted in score 2 [11]. The Platelet-to-Lymphocyte Ratio (PLR), derived by dividing the platelet count by the absolute lymphocyte count, had a threshold of ≤123 [12]. The HALP score was calculated using the formula hemoglobin × albumin × lymphocytes/platelet count, with a high HALP defined as ≥26 [13]. A high Lymphocyte-to-Monocyte Ratio (LMR) was set at ≥2.6 [14], whereas a high Monocyte-to-Lymphocyte Ratio (MLR) was defined as ≥0.367 [15]. The Systemic Inflammation Index (SII) was computed as platelet count × neutrophil count/lymphocyte count, with a high SII defined as ≥479 [16]. The Pan-immuno-inflammation value (PIV) was calculated as neutrophil count × platelet count × monocyte count/lymphocyte count, with a cutoff value defined as >390 [17]. The Prognostic Nutritional Index (PNI), calculated as serum albumin (g/L) + 5 × total lymphocyte count (10^9^/L), was defined as ≤31 [18]. The CAR (C-reactive Protein-to-Albumin Ratio) inflammation score is obtained by dividing the level of C-reactive Protein (CRP) by the level of albumin in the blood with a threshold of >0.742 [19].

### 2.3. Statistical Analysis 

Statistical analyses were conducted using R statistical software, version 4.1.2, from the Institute for Statistics and Mathematics in Vienna, Austria. In the initial step, ischemic stroke patients without atrial fibrillation were divided into groups based on the presence of LAT. Frequencies and percentages were used to present categorical data. The chi-square test was employed to compare categorical variables between groups. Continuous variables with normal distributions were expressed as mean (standard deviation), while those with non-normal distributions were presented as median (interquartile range [IQR]). The Kolmogorov–Smirnov test and histogram analysis were used to assess the normality of the variables. For group comparisons, an independent Student’s *t*-test was used for normally distributed variables, while the Mann–Whitney U test was applied for non-normally distributed variables. To identify independent factors associated with LAT, univariate and multivariate Cox regression analyses were performed using the enter method. Variables with a *p*-value < 0.2 in the univariate analysis were included in the multivariate Cox regression analysis to determine independent predictors of LAT. Non-parametric Receiver Operating Characteristic (ROC) curves were utilized to analyze the optimal cutoff value for LAT presence, providing the best sensitivity and specificity. All data were presented as hazard ratios (HR) with 95% confidence intervals (CI). A *p*-value ≤ 0.05 was considered statistically significant. G*Power 3.1 was used to estimate the required sample size; 95% power; a = 0.05. Cohen’s d (effect size) = 0.5 was selected and the minimum number of patients meeting the required characteristics for the pre-study Mann–Whitney U test was found to be 31 + 31 = 62.

## 3. Results 

### 3.1. Patients Characteristics

In the present study, the mean age was 68 ± 13 years and 175 (58%) of the patients were male. Among 303 patients, 34 had a diagnosis of LAT at the time of TEE examination. We classified the study cohort into two groups: patients with LAT (+) and those without LAT (−). Table 1 summarizes the patient baseline characteristics. According to the differences in age and gender, no significant difference was detected between the LAT (+) and LAT (−) patients. The study results showed that patients who had LAT had a higher prevalence of diabetes and a history of cancer.

Table 2 compares laboratory values and echocardiographic findings. Hemoglobin, hematocrit albumin, and cholesterol were found to be statistically significantly lower in the LAT (+) patients. INR, CRP, troponin, and Brain Natriuretic Peptide (BNP) were observed to be statistically significantly higher in the LAT (+) patients. Echocardiographically, higher Left Atrium Anterior–Posterior Dimension (LAAP-D) and Systolic Pulmonary Artery Pressure (SPAP) were found to be greater in the LAT (+) patients.

The independent predictors of LAT were identified using both univariate and multivariate logistic regression analyses. Initially, univariate logistic regression was performed to assess the association between each variable and the presence of LAT (Supplementary Table S1). Variables with a *p*-value < 0.2 in univariate analysis were included in the multivariate model, and results were reported as odds ratios (OR) with 95% confidence intervals (CI). Table 3 presents a multivariable logistic regression analysis to determine the independent variables for LAT. According to this analysis, CAR (OR: 2.7001, 95% CI: 1.3874–5.2547, *p* = 0.003), BNP (OR: 1.1062, 95% CI: 1.0014–1.2219, *p* = 0.046), LAAP-D (OR: 1.2258, 95% CI: 1.1367–1.3220, *p* = 0.014), and SPAP (OR: 1.0857, 95% CI: 1.0005–1.1782, *p* = 0.048) were independent predictors for LAT in ischemic stroke without AF patients. The Receiver Operating Characteristic (ROC) analysis demonstrated the area under the curve (AUC) for the performance of independent predictors in predicting LAT in ischemic stroke without atrial fibrillation (Supplementary Figure S1).

### 3.2. Comparisons of Inflammation Scores

In patients undergoing TEE, the HALP score (*p* = 0.018), PNI (*p* = 0.022), and CAR (<0.001) were the inflammation scores most strongly associated with LAT when evaluated separately. Other inflammation markers, including the NLR, LMR, MLR, SII, PIV score, and Naples scores were inferior to predict LAT in ischemic stroke (Table 2). Based on the results of the multivariate regression analysis, the CAR (0.003), (OR: 2.7001 (95% CI: (1.3874–5.2547)) emerged as the only independent predictor among the inflammation scores. The CAR consistently demonstrated the highest prognostic value for LAT compared to the other inflammation scores in ischemic stroke patients (Table 3). 

**Figure 1:**
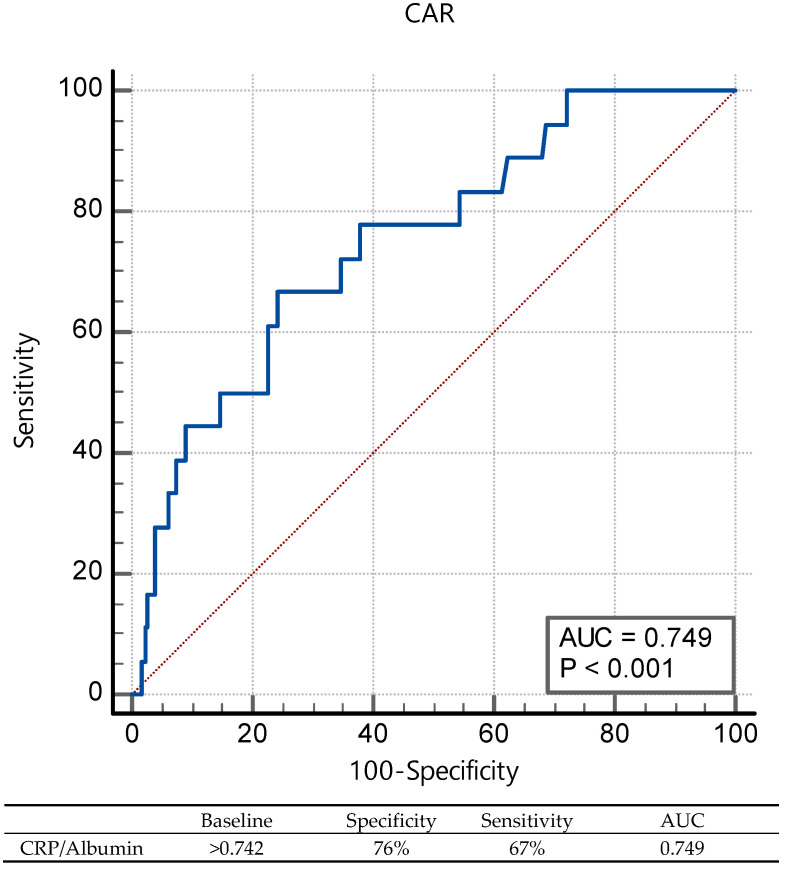
Performance of CAR for predicting left atrial thrombus in ischemic stroke without atrial fibrillation.

The Receiver Operating Characteristic (ROC) analysis demonstrated an area under the curve (AUC) of 0.749 (*p* < 0.001) for the CAR score and was statistically significant in the prediction of LAT in ischemic stroke patients (Figure 1).

## 4. Discussion

Effective predictive markers in clinical practice require being readily accessible, consistently reproducible, and, above all, instrumental in enhancing clinical decision-making for patient care. Inflammation scores, derived from commonly measured inflammation markers, have gained attention due to their availability and ease of use, as these standard blood tests generally demonstrate high reproducibility [20]. However, it is essential to determine which of these scores offers the greatest clinical utility, particularly in predicting LAT in ischemic stroke patients without AF. To the best of our knowledge, this study is the first to address this topic.

AF is the most clearly established risk factor for cardio-embolic stroke. Nonetheless, a previous case–control study using TEE revealed that thrombus formation can also occur in individuals with normal sinus rhythm due to reduced flow velocities in the left atrial appendage (LAA). In the literature, studies have found that the frequency of LAT in ischemic stroke patients without AF is 6.2–9.3% [21,22]. In our study, the frequency of LAT was 10.3% (34 out of 303 patients). We analyzed ten inflammation scores that have been previously explored in the literature. The results show that among systemic inflammation scores, the HALP, CAR, and PNI scores were significant predictors of LAT in patients with ischemic stroke. Moreover, multivariable logistic regression analysis demonstrated that among the inflammation scores, only the CAR score was an independent predictor for LAT in stroke patients.

The CAR has emerged as a promising marker of systemic inflammation, offering enhanced sensitivity and specificity compared to C-reactive Protein (CRP) or serum albumin levels alone [23,24]. CRP, an acute-phase protein, is generated in response to cytokine activation due to infection, ischemia, trauma, and other inflammatory stimuli. Elevated CRP levels are widely linked with prognosis and mortality in cardiovascular conditions. Similarly, low serum albumin has been associated with poor prognosis and increased mortality, underscoring the value of CAR as a combined marker for assessing inflammatory status [25]. Recent studies endorse CAR as a valuable marker in cardiovascular disease, demonstrating associations with no-reflow phenomena and acute stent thrombosis [26]. Another study investigated the correlation between CAR and left ventricular thrombosis in patients with anterior STEMI treated via percutaneous intervention [27]. Huang et al. found that CAR is linked to stroke-associated pneumonia and early clinical outcomes in patients with acute ischemic stroke [28]. Although no previous studies have examined the relationship between CAR and LAT, our results are consistent with previous studies, showing that an increased thrombus burden is associated with a higher CAR. The CAR score can statistically significantly predict LAT in ischemic stroke patients without AF.

Our study revealed that high-sensitivity C-reactive Protein (hsCRP) levels were significantly elevated in stroke patients with LAT [29]. Elevated hsCRP levels reflect a pro-inflammatory state that may promote endothelial dysfunction, hypercoagulability, and thrombus development in the left atrium (LA) [30]. Recent studies suggest that hsCRP is an important biomarker for predicting cardiovascular diseases. It predicts incident myocardial infarction, stroke, peripheral arterial disease, and sudden cardiac death among healthy individuals with no history of cardiovascular disease, as well as recurrent events and death in patients with acute or stable coronary syndromes. hsCRP provides additional prognostic value at all levels of cholesterol, Framingham coronary risk score, the severity of the metabolic syndrome, and blood pressure in individuals with and without subclinical atherosclerosis [31,32]. In our study, low albumin levels were found to be associated with the presence of LAT. Serum albumin levels are inversely proportional to the extent of inflammation, primarily due to reduced albumin synthesis by the liver during inflammatory processes and the secondary effects of cytokine production, including TNF-α and IL-6. Albumin levels below 35 g/L are widely recognized as indicative of chronic inflammation [33]. Previous studies have demonstrated that patients with albumin levels between 30 and 39.9 g/L have a 1.5-fold higher adjusted risk of thromboembolism compared to those with levels ≥ 40 g/L, with the risk increasing further at lower albumin levels [34]. In our cohort, the albumin levels of both groups were consistent with the findings reported in the literature.

In this study, BNP is identified as another independent predictor for LAT in stroke without AF patients. BNP is a cardiac hormone that exerts antifibrotic effects in the heart and is generated following the cleavage of its precursor, pro-BNP. It is released from myocytes due to stretch stimulation in response to increased wall tension and volume or pressure overload, as well as during periods of hemodynamic stress. This relationship between BNP and atrial dilatation accounts for the significant correlation between elevated BNP/NT-proBNP levels and the occurrence of cardio-embolic stroke. Increased pro-BNP concentrations have been noted in various conditions involving atrial abnormalities, such as atrial dilatation and left atrial appendage dysfunction, which increase the risk of ischemic stroke [35,36,37]. The BNP level is associated with left ventricular filling pressure, making it a potential marker for predicting LAT [38]. Research has shown that BNP can independently predict LAT, separate from established predictors like the CHADS2 and CHA2DS2-VASc scores, which are commonly used to assess the risk of LA appendage thrombus (LAAT) and systemic embolization in AF [39]. Additionally, a recent retrospective study involving 524 non-anticoagulated AF patients identified BNP as an independent predictor of LAAT, with a plasma BNP level of 251 pg/mL serving as a valuable threshold for prediction [40]. Another study found that, in patients without significant valvular heart disease, BNP levels were more strongly correlated with left ventricular filling indices than other echocardiographic measures. Notably, the left atrial volume index emerged as the strongest correlate of BNP and a key predictor of thrombus formation [41].

D-dimer is a plasma biomarker derived from cross-linked fibrin, indicating active coagulation and fibrinolysis [42]. Several studies have investigated the relationship between D-dimer levels and the presence of LAT in stroke patients [43]. In our cohort, D-dimer levels were elevated in both groups but did not show differentiation between them. While D-dimer can be elevated in patients with stroke, its levels may not consistently differentiate between those with and without LAT. Sierra et al. found that higher cutoff points for D-dimer did not rule out the presence of LAT [44]. Wan et al. revealed that although high D-dimer levels were associated with LAT, the sensitivity and specificity were moderate, suggesting that D-dimer alone may not be a reliable marker for LAT detection [45].

Echocardiographic evaluation is applied in patients who exhibited significantly higher LAAP-D and SPAP, both of which were identified as independent predictors. Interstitial fibrosis, driven by elevated left-sided filling pressures, has been demonstrated to contribute to increased left atrial (LA) stiffness and compromised LA contractility. Consequently, this leads to elevated pulmonary pressures, resulting in tricuspid valve regurgitation and further elevation of SPAP. In accordance with these pathophysiological mechanisms, it was observed that patients with LAT had larger atrial dimensions and exhibited significant tricuspid regurgitation, which also correlated with increased SPAP [46,47]. The association between LA diameter and an increased risk of LAT was first identified. LA diameter has been linked to stroke risk, with Lee et al. demonstrating a correlation between LA diameter and stroke history in patients with a low CHA2DS2-VASc score [48]. LA dilation is known to predispose patients to various complications, including thrombus formation, thromboembolic events, hemodynamic disturbances, and sudden death. An enlarged LA often leads to blood stasis, which facilitates thrombus development [49]. The risk of thromboembolism rises with increasing LA size, even in patients receiving anticoagulation therapy. Previous studies have reported that a direct relationship was observed between thrombus occurrence and LA size [50].

We considered that our findings may be useful in terms of clinical applicability. A risk stratification system that enables identifying the patients who would require further TEE examination could be performed based on our study results. Particularly, ischemic stroke patients with sinus rhythm who had high CAR scores had high LAAP-D and SPAP on TTE and higher BNP values, representing a high-risk group for LAT. Therefore, these patients should undergo further TEE examination to verify the possibility of a cardio-embolic source.

## 5. Conclusions

According to our cohort, CAR was identified as the optimal score among ten inflammation scores for predicting LAT in ischemic stroke patients without AF. As far as we know, there is no study on this subject in the literature.

## 6. Limitation

Our study has several limitations. The primary limitation is its retrospective design. Retrospective studies, while valuable for generating hypotheses and analyzing existing data, often face significant limitations, including inherent biases and confounding variables. Despite applying multivariate analysis to identify independent predictors of LAT, some unmeasured confounders may still be present. Prospective studies, by design, allow for the collection of data in real time and enable researchers to implement rigorous controls over variables, thereby enhancing the validity of the findings. Designing this as a multicenter, prospective study will enhance the study’s strength and its applicability in clinical practice. Another limitation of this study is the small sample size. Small sample sizes in studies can lead to decreased statistical power, limiting the generalizability of the results. In order to minimize these limitations, it is essential that studies are conducted in a multicenter, multi-sample size, participatory, and robust methodology. Additionally, all scores were calculated based solely on admission laboratory values. 

## 7. Future Directions

Future research should focus on validating the utility of inflammation scores as predictive tools for LAT in broader patient populations. Multicenter prospective studies with diverse cohorts, including patients with different stroke subtypes and varying comorbidities, are necessary to establish the generalizability of these scores. Furthermore, leveraging advanced artificial intelligence (AI) methods to integrate these inflammatory markers with advanced imaging techniques and other clinical risk factors—such as atrial size, ventricular function, and biomarkers like BNP, D-dimer, and hsCRP—could enhance the accuracy of LAT prediction. The development of a comprehensive AI-based risk stratification model incorporating inflammation scores may guide clinical decision-making, particularly for anticoagulation management in stroke patients without atrial fibrillation. Finally, exploring the mechanistic relationship between systemic inflammation and thrombus formation in the LA could provide novel insights, potentially leading to targeted therapeutic strategies.

## Figures and Tables

**Table 1:** Baseline demographic features of patients.

	All Patients (n = 303)	Left Atrial Thrombosis (−) (n = 269)	Left Atrial Thrombosis (+) (n = 34)	*p* Value
Age	68.7 (56–80)	69 (58–80)	66 (56–79)	0.433
Gender, male (%)	175 (58%)	155 (58%)	20 (59%)	0.732
HT	218 (72%)	197 (73.5%)	21 (61.8%)	0.150
DM	109 (36%)	103 (38.3%)	6 (17.6%)	0.018
COPD	20 (6.6%)	20 (7.7%)	0 (0%)	0.388
Dementia	17 (5.6%)	17 (8.4%)	0 (0%)	0.139
Cancer	13 (4.2%)	9 (4.4%)	4 (16.7%)	0.015
CKD	40 (13.2%)	34 (12.7%)	6 (17.6%)	0.422
Hospitalization	5.4 (3–19)	5 (3–9)	8.5 (4–19)	0.053
In-Hospital Mortality	19 (6.2%)	15 (7.4%)	4 (16.7%)	0.118

Abbreviations: CKD: chronic kidney disease, COPD: chronic obstructive pulmonary disease, DM: diabetes mellitus, HT: hypertension.

**Table 2:** Examination of laboratory, echocardiography, and indices with Mann–Whitney U test.

Laboratory	Left Atrial Thrombosis (−)	Left Atrial Thrombosis (+)	*p* Value
WBC (10^3^/µL)	8.25 (6.8–10)	8.4 (6.5–11.5)	0.722
HGB (g/dL)	12.8 (11.4–14.4)	11.7 (10.3–13.5)	0.011
Hematocrit	38.5 (34.7–42.7)	35.8 (32.6–40.1)	0.032
Platelet (10^3^/µL)	229 (184–285)	255 (194–333)	0.222
Creatinine (mg/dL)	1.09 (0.9–1.3)	1.18 (0.9–1.47)	0.404
AST (U/L)	20 (16–28)	21 (16–27)	0.843
ALT(U/L)	19 (14–28)	17 (10–27)	0.266
Glucose (mg/dL)	110 (89–146)	106 (91–140)	0.888
hsCRP (mg/dL)	5.8 (0–20.5)	27 (6.7–83)	<0.001
Albumin (g/L)	38 (33–41)	33.5 (29.5–38)	0.032
Troponin (ng/dL)	6.5 (2–38.2)	18 (11–58)	0.014
BNP (pg/mL)	288 (72–936)	1322 (496–4753)	<0.001
D-dimer (ng/mL)	900 (172–5290)	1203 (545–3465)	0.873
Total cholesterol (mg/dL)	186 (163–216)	173 (127–200)	0.025
LDL (mg/dL)	115 (91–144)	106 (84–142)	0.262
HDL (mg/dL)	40 (33–50)	38 (29–40)	0.041
Triglyceride (mg/dL)	130 (95–193)	114 (83–150)	0.123
Echocardiography			
EF%	60 (50–60)	60 (45–66)	0.817
LVDD	46.6 ± 6.5	52.2 ± 13	0.022
LVSD	37 ± 9.4	40.8 ± 12	0.296
LAAP-D	38.2 ± 5.4	46.5 ± 8.2	<0.001
SPAP	35 (28–41)	50 (35–55)	<0.001
Indices			*p* value
NLR	2.55 (1.75–4.29)	2.88 (2.20–4.44)	0.245
LMR	3.88 (2.69–5.13)	3.40 (2.41–4.98)	0.537
MLR	0.26 (0.19–0.37)	0.29 (0.20–0.41)	0.537
PLR	123 (88–164)	131 (100–206)	0.136
PIV index	299 (168–598)	365 (220–721)	0.218
PNI	47.5 (41.6–52.2)	43 (38–47)	0.022
SII	605.7 (387.4–1013.1)	696 (458–1518)	0.142
HALP score	38.5 (23.8–58.2)	21 (11.4–42)	0.018
CAR	0.19 (0–0.74)	1.07 (0.28–3.32)	<0.001
Naples score	2 (1–3)	3 (1.75–4)	0.319

Abbreviations: echocardiography: EF: Ejection Fraction, LAAP-D: Left Atrium Anterior–Posterior Dimension, LVDD: Left Ventricle Internal Diastolic Dimension, LVSD: Left Ventricle Internal Systolic Dimension, SPAP: Systolic Pulmonary Artery Pressure. Indices: CAR: C-reactive Protein–Albumin Ratio, HALP: hemoglobin, albumin, lymphocyte and platelet, NLR: Neutrophil–to-Lymphocyte ratio, LMR: Lymphocyte-to-Monocyte Ratio, MLR: Monocyte-to-Lymphocyte Ratio, SII: Systemic Inflammation Index, PLR: Platelet-to-Lymphocyte ratio, PIV: Pan-immuno-inflammation value, PNI: Prognostic Nutritional Index.

**Table 3:** Multivariable logistic regression for detecting the independent associations of variables with left atrial thrombus.

Variables	OR	95% CI	*p*-Value
CAR	2.7001	1.3874–5.2547	0.003
BNP	1.1062	1.0014–1.2219	0.046
SPAP	1.0857	1.0005–1.1782	0.048
LAAP-D	1.2258	1.1367–1.3220	0.014

Abbreviations: BNP: Brain Natriuretic Peptide, CAR: C-reactive Protein–Albumin Ratio, LAAP-D: Left Atrium Anterior–Posterior Dimension, SPAP: Systolic Pulmonary Artery Pressure.

## Data Availability

The datasets generated during and/or analyzed during the current study are available from the corresponding author upon reasonable request. Due to legal and ethical concerns, publication of the data is not preferred.

## Supplementary Materials

The following supporting information can be downloaded at: https://www.mdpi.com/article/10.3390/medicina60122046/s1, Figure S1: predictive performance of CAR, BNP, SPAP, and LAAP-D to detect left atrial thrombus in ischemic stroke without atrial fibrillation; Table S1: univariable logistic regression for independent variables associated with left atrial thrombus.
