# Supplementary material for: Predictive Value of Inflammatory Scores for Left Atrium Thrombosis in Ischemic Stroke Without Atrial Fibrillation
Source: Medicina (Kaunas). 2024 Dec 12;60(12):2046. doi: 10.3390/medicina60122046 (PMC11677452; doi:10.3390/medicina60122046)
Supplement: Supplementary file 1 [file medicina-60-02046-s001.zip › medicina-3322882-supplementary.pdf]

Supplementary Table S1: Univariable logistic regression for independent variables associated with left atrial thrombus

	Univariate logistic regression		
	OR	95%CI	P value
Diabetes mellitus	0.3454	0.1383-0.8626	**0.023**
Cancer	4.3111	1.2173-15.2680	**0.023**
LAAP-D	1.7209	1.166 – 2.704	**<0.001**
Systolic pulmonary artery pressure	1.0766	1.0291-1.262	**0.0014**
Hemoglobin	0.9953	0.8759-1,1311	0.943
Hematocrit	0.9493	0.8966-1.0051	0.074
BNP	1.1071	1.0555-1.1611	**<0.001**
High-sensitive CRP	1.0095	1.0032-1.0159	**0.003**
Albumin	0.9291	0.8638-0.9993	**0.048**
Troponin	0.9992	0.9972-1.0013	0.461
Total cholesterol	0.9854	0.9741-0.9968	**0.012**
High density lipoprotein	0.9611	0.9228-1.0011	0.056
Triglyceride	0.9927	0.9849-1.006	0.068
PLR	1.0045	1.0006-1.0084	**0.023**
CAR	1.2405	1.0536-1.4607	**0.009**
PIV index	1.0003	1.0000-1.0007	0.083
PNI	0.9408	0.8888-0.9958	**0.035**
HALP	0.9884	0.9690-1.0082	0.248

Abbreviations: **LAAP-D**: Left atrial anterior-posterior diameter, **BNP:** brain natriuretic peptide, **CAR:** C-Reactive protein-albumin ratio, **HALP:** Hemoglobin, Albumin, Lymphocyte and Platelet, **PIV:** Pan-immuno-inflammation value, **PNI:** Prognostic nutritional index, **SPAP**: Systolic pulmonary artery pressure

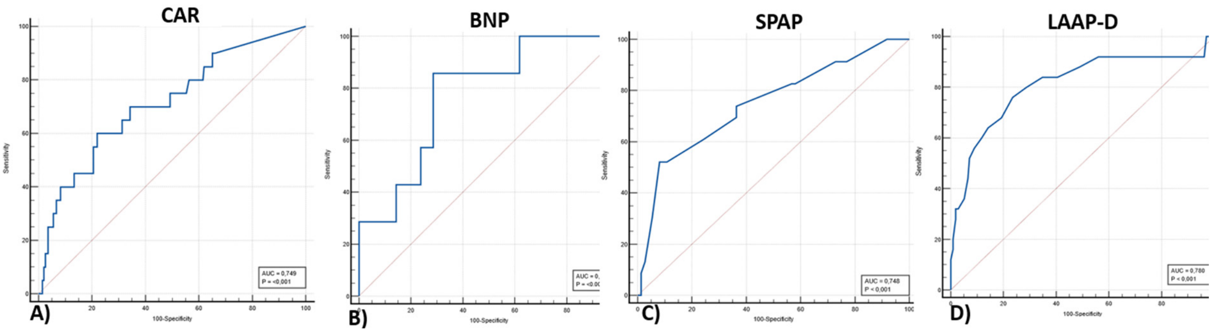

	Cut-off	Specificity	Sensitivity	AUC
CAR	>0.742	76%	67%	0.749
BNP	>479	71%	86%	0.776
SPAP	>40	76%	61%	0.748
LAAP-D	>41	78%	77%	0.780

Supplementary Figure S1: Predictive performance of CAR, BNP, SPAP AND LAAP-D to detect left atrial thrombus in ischemic stroke without atrial fibrillation.

Abbreviations: **CAR:** C-Reactive protein-albumin ratio, **BNP:** brain natriuretic peptide, **SPAP**: Systolic pulmonary artery pressure and **LAAP-D**: Left atrial anterior-posterior diameter
